# The Clinical Significance of Warm Autoantibodies during Pregnancy

**DOI:** 10.1111/tme.70008

**Published:** 2025-08-07

**Authors:** Emmanuel A. Fadeyi, Joshua Cox‐Jones, Amit K. Saha, Daniel Katz, Bettina Turner, Christina S. Warren, Gregory J. Pomper

**Affiliations:** ^1^ Department of Pathology Wake Forest University School of Medicine Winston‐Salem North Carolina USA

**Keywords:** hemolytic disease of fetus and newborn, immunoglobulin g antibodies, pregnancy, warm autoantibody, warm autoimmune hemolytic anemia

## Abstract

**Objectives:**

The objective of the current study is to determine the clinical significance of a warm autoantibody detected in patients during pregnancy.

**Background:**

There are few published studies concerning the clinical significance of warm autoantibodies during pregnancy. The risk to the fetus is determined by the IgG autoantibody's ability to cross the placental barrier.

**Materials and Methods:**

Existing data of all obstetric patients who had a positive antibody screen with a warm autoantibody diagnosis during their pregnancy in the last 7 years from August 2016 to October 2023 were reviewed. If positive, a direct antiglobulin test (DAT) and an eluate were performed. Statistical analysis was performed to determine the clinical significance of warm autoantibody in pregnant patients. Data collected included blood type, race, age, BMI, the most recent hemoglobin before delivery, and gestation in weeks.

**Results:**

Between August 2016 and October 2023, 23 510 pregnant patients had blood type and antibody screen completed at our institution. A total of 812 (3.5%) patients had a positive antibody screen. Only 16 (<2.0%) patients had a positive DAT and eluate confirmation of a warm autoantibody. None of the 16 patients had a previous history of warm autoantibody. 14/16 mothers did not experience an AIHA or HDFN in the newborns.

**Conclusion:**

Pregnancy‐induced warm autoantibody appears to be harmless for most mothers and their babies. The detection of a warm autoantibody in pregnancy may reflect a potential risk for both the mother and the child; however, on follow up, there were no clinical complications associated with warm autoantibodies in our patient cohort.

## 1 INTRODUCTION

AIHA is caused by autoantibodies that react with self‐red blood cells (RBCs) and cause them to be destroyed. Warm AIHA, due to antibodies that are active at body temperature, is the most common type of AIHA. Antibodies to RBCs may show various effects in vivo on circulating RBCs compared to their reactivity with RBCs in serological testing. In addition, the clinical significance of antibodies detected serologically often depends on the clinical condition of the individual patient. Some patients may develop a severe hemolytic transfusion reaction due to an incompatible RBC transfusion, whereas other patients may develop only mild or no reactions under identical serological findings.^1^, ^2^ The clinical significance of warm autoantibody in pregnancy and the effect on the fetus have not been well described.^3^, ^4^, ^5^, ^6^, ^7^, ^8^, ^9^, ^10^ The DAT positive with warm autoantibody alone does not define AIHA. According to the international consensus meeting published in^11^ Jager et al. AIHA is hemolytic anemia caused by the destruction of RBCs through autoantibodies directed against antigens on their surface and the diagnostic criteria of AIHA is ‘evidence for hemolysis accompanied by a positive direct antiglobulin test (DAT) and exclusion of alternative causes, such as a delayed hemolytic transfusion reaction’. Therefore, having a positive DAT with warm autoantibody detected in the indirect antiglobulin test alone does not define hemolysis or AIHA.

Autoantibodies, particularly warm‐reactive immunoglobulin G (IgG) antibodies, most frequently display pan‐reactivity against all RBCs; and though they may complicate identification of underlying alloantibodies, they are typically easily distinguishable from most alloantibodies because they lack antigen specificity. In rare cases, warm autoantibodies may display apparent in vitro specificity for a particular RBC antigen, most frequently Rh antigens.^12^, ^13^ The development of RBC autoantibodies does not require previous exposure to RBC antigens and may be idiopathic or occur secondary to a variety of conditions, including underlying malignancies, infections, or autoimmune disorders.^2^, ^14^, ^15^, ^16^

Warm AIHA may be exacerbated during pregnancy, and in rare instances, the maternal autoantibody may cross the placenta and affect the fetus or newborn.^3^, ^4^, ^5^, ^17^ Since warm autoantibody is rare in pregnancy, little is known about its characteristics, management, and maternal‐fetal outcomes.

Correlation with MCA Doppler study is usually recommended if clinically indicated. The objective of the current study is to determine the clinical significance of a warm autoantibody detected in patients during pregnancy.

## 2 STUDY DESIGN AND METHODS

All obstetrics patient's data who were seen at any of the Atrium Health Wake Forest Baptist Network including outpatient clinics from August 1st, 2016, to October 31, 2023, were collected from the hospital electronic medical records. All non‐obstetrics female patients who are not currently pregnant were excluded. Blood Bank software was used to collect data of all pregnant patients who had an ABO Rh positive antibody screen and a diagnosis of warm autoantibody during the 7‐year period. The ABO Rh and antibody screen were obtained as part of the prenatal work up and continuing evaluation of hemolytic risk if positive during pregnancy. This will be considered a high‐risk pregnancy.

The medical records of the patients with warm autoantibody and their newborns were reviewed for clinical diagnosis, laboratory data, signs of hemolysis, and symptoms of autoimmune hemolytic anemia. A clinical diagnosis of HDFN or autoimmune hemolytic anemia would be considered present if any of the following were present in the medical record: HDFN in the newborn was regarded as evidence of hemolysis; a positive DAT with maternal antibody either present in plasma or eluted from neonatal red blood cells; jaundice; elevated indirect bilirubin; decreased hemoglobin; and high LDH requiring phototherapy or exchange transfusion. Hemolytic anemia in the mother was regarded as evidence of hemolysis; a decreased hemoglobin; high LDH; high indirect bilirubin; and reticulocytes with decreased haptoglobin.

Commercial red cell antibody screen (Ortho Clinical Diagnostics) and identification panels using gel technique were used. Plasma gel antibody screen at (37°C) was performed in all cases and, if positive, a direct antiglobulin test; if positive, an eluate was performed. Reagent RBCs and antisera used for the standard tube DAT were from Immucor (Norcross, GA). Other reagent RBCs were from Ortho‐Clinical Diagnostics Inc. (Raritan, NJ) and from Bio‐Rad Laboratories, Inc. (Dreicich, Germany). The laboratory completed the antibody panel using a Gel Micro Typing System card (Ortho‐Clinical Diagnostics). Elution studies were performed using the Elukit (Immucor). Adsorptions using low ionic strength solution as additive were performed with the gel antibody panel on all the patients to rule out a concurrent alloantibody. Wake Forest University Health Sciences' Institutional Review Board approved this data collection protocol.

## 3 STATISTICAL ANALYSIS

To determine the clinical significance of warm autoantibody in pregnant patients with a positive antibody screen. Data will be analysed using descriptive statistics to compare the results of positive warm autoantibody during pregnancy with evidence of AIHA in the mother or hemolytic disease of the fetus and newborn (HDFN) during pregnancy and after delivery. Variables associated with positive warm autoantibody were included in a multivariable logistic regression model. Independent variables that were chosen a priori for inclusion in the multivariable model were race, blood type, age, BMI, most recent value of haemoglobin before delivery and gestational age in weeks. A two‐tailed *p*‐value of <0.05 was considered statistically significant. We computed the odds ratios (ORs) following multivariable logistic regression. All statistics were reported as point values with 95% confidence intervals (CIs). Data were analysed using SAS software version 9.4 (SAS Institute, Cary, NC).

## 4 RESULTS

Between August 2016 and October 2023, 23 510 pregnant patients had blood type and antibody screen completed at our institution. A total of 812 (3.5%) patients had a positive antibody screen. 16 (1.9%) of these patients had a positive DAT and eluate confirmation of a warm autoantibody. The remaining 796 patients had alloantibodies. All the DAT results showed both immunoglobulin G (IgG) and polyspecific positive reactions, while only seven of the patients showed an additional positive complement (C3d) reaction. All the antibody screens, antibody identification panels, and eluates demonstrated pan‐agglutinin reactions with 3+ or greater in agglutination strength. Results of the allo adsorptions were negative in all the 16 patients for concurrent alloantibody. Since IgG antibodies are not efficient in complement activation, most warm autoantibodies usually demonstrate only IgG specificity and may or may not demonstrate reactivity with complement. Every warm autoantibody was detected during pregnancy and not prior to the first prenatal antibody screen. Alloantibodies detected in the remaining 796 patients included anti‐D, anti‐E, anti‐c, and anti‐K. All these patients were excluded from our follow up. Of the 812 patients with positive antibody screens, 123 were RhD positive. All 16 patients with confirmed warm autoantibody were RhD positive (16/123). The 16 patients with a detected warm autoantibody had no previous history of warm autoantibody, nor did these 16 patients have any alloantibodies detected during pregnancy. Ten of the 16 patients had more than one previous pregnancy, with the remaining 6 noted to be in their first pregnancy (See Table 3). All the 16 patients did not have a previous history of positive antibody screens. The median age and the most recent hemoglobin before delivery were 21 years old and 10.9 g/dL, respectively. The median BMI and the gestational age of the pregnancies were 26.3 and 37 weeks, respectively (See Table 1). No evidence of AIHA or HDFN was present in the mother. Two patients could not be followed after delivery. One of these mothers was lost to follow up at 14 weeks gestation, and the other patient had a spontaneous abortion at 7 weeks (See Table 3). All the remaining 14 newborns at birth had no clinical evidence of hemolysis such as jaundice or the need for phototherapy; the appearances of their body and extremities were pink with no cyanosis and no DAT were obtained due to no medical reasons necessitating hemolytic work up (See Table 3). Only four of the neonates had ABO Rh type completed, and one had ABO incompatibility with her mother. The mother was group B positive, and the neonate was group O positive. All the 14 newborns were discharged in stable conditions. A logistic regression analysis was performed for predictors of positive warm autoantibody following predictor variables: race, blood type, age, BMI, most recent value of hemoglobin before delivery, and gestational age in weeks. Of these covariates, age (OR: 0.846 95% CI: 0.768–0.931, *p*‐value <0.001) and BMI (OR: 0.843 95% CI: 0.767–0.927, *p*‐value <0.001) are predictors of positive warm autoantibody (Table 2). Table 4 described the past medical history and any pregnancy complications of the 14 cases of mothers with warm autoantibody.

**TABLE 1:** Patient demographics, gestational age and hemoglobin before delivery.

	Positive antibody screen	Warm autoantibody only
Number of patients, 812	796 (98%)	16 (2%)
Race		
White or Caucasian	600 (74%)	6 (0.74%)
Black or African American	117 (14%)	6 (0.74%)
Other (Hispanic, Latino and non‐Hispanic, and Latino)	95 (12%)	4 (0.52%)
ABO group		
A	331 (41%)	6 (0.74%)
AB	26 (3%)	1 (0.12%)
B	80 (10%)	3 (0.40%)
O	375 (46%)	6 (0.74%)
Rh type		
NEG	689 (85%)	0 (0%)
POS	123 (15%)	16 (2%)
Age (Median, IQR)	29, [24,34]	21 [19,27]
BMI (Median, IQR)	31.18 [27.31, 36.61]	26.3 [21.9, 31.5]
Most recent value of hemoglobin before delivery (Median, IQR)	11.7 [10.9, 12.5]	10.9 [10.3, 12.01]
Gestational age (w), (Median, IQR)	38 [36, 39]	37 [36, 38.5]

**TABLE 2:** Univariate analysis, odds ratio of warm autoantibody.

Covariate	Odds ratio	95% CI for Odds ratio		*p*‐Value
		Lower	Upper	
Race				
White or Caucasian	Reference			
Black or African American	5.13	1.63	16.18	0.01
Other	4.21	1.17	15.2	0.03
Hispanic, Latino or Spanish	0.28	0.09	0.9	0.03
ABO group				
A	1.13	0.36	3.54	0.83
AB	2.4	0.28	10.66	0.43
B	2.34	0.57	9.54	0.24
O	Reference			
Age	**0.85**	**0.77**	**0.93**	**<0.001**
BMI	**0.84**	**0.77**	**0.93**	**<0.001**
Most recent value of hemoglobin before delivery	0.79	0.56	1.12	0.19
Gestational age in weeks	1.01	0.84	1.22	0.89

*Note*: Statistical significance and are both predictors of warm autoantibody.

Four of the 16 patients had middle cerebral artery (MCA) Doppler studies that demonstrated ranges consistent with minimal risk for anaemia (Table 3). In the three mothers, the MCA Doppler was performed due to the presence of warm autoantibody and in one mother due to intra‐uterine growth restriction. There were no clinical features or symptoms of warm AIHA in the mothers. Review of delivery records of all our 14 cases showed an average birth age of 37 weeks, range of (26–39) weeks with unremarkable physical examination and no signs of haemolytic disease of the newborn at birth. Follow‐up during the observation period demonstrated an absence of clinical signs and symptoms of haemolysis and no diagnosis of AIHA in the mothers. Our study did not show clinical significance in pregnant patients with a serologically detected warm autoantibody and development of AIHA or HDFN in the mother.

**TABLE 3:** Serology, gestational ages, MCA Dopplers, hematologic status of the newborns in cases of warm autoantibody.

Case	IAT	Plasma Ab panel	IgG‐DAT	C3d‐DAT	Eluate Ab panel	GA/GP	MCA Doppler (MoM)	Total Bilirubin	HB	Apgar scores	Type of delivery	HDFN
1	Pos	Pos	Pos	Neg	Pos	39/G3P2	N/A	None	None	8, 9	Vaginal	No
2	Pos	Pos	Pos	Neg	Pos	36/G1P0	1.13	None	None	8, 9	Vaginal	No
3	Pos	Pos	Pos	Pos	Pos	36/G1P0	N/A	None	None	4, 7	Cesarean	No
4	Pos	Pos	Pos	Neg	Pos	38/G3P2	N/A	None	None	8, 9	Cesarean	No
5	Pos	Pos	Pos	Neg	Pos	38/G4P1	N/A	5.0	20.8	9, 9	Vaginal	No
6	Pos	Pos	Pos	Neg	Pos	39/G3P1	N/A	8.5	15.1	9, 9	Cesarean	No
7	Pos	Pos	Pos	Neg	Pos	38/G3P2	N/A	None	None	8, 9	Vaginal	No
8	Pos	Pos	Pos	Neg	Pos	14/G1P0	N/A	N/A	N/A	N/A	N/A	N/A
9	Pos	Pos	Pos	Pos	Pos	36/G1P0	1.19	None	None	7, 9	Vaginal	No
10	Pos	Pos	Pos	Neg	Pos	7/G2P1	N/A	N/A	N/A	N/A	N/A	N/A
11	Pos	Pos	Pos	Pos	Pos	39/G4P3	N/A	6.3	None	8, 9	Vaginal	No
12	Pos	Pos	Pos	Pos	Pos	26/G5P3	1.09	7.5	13.1	6, 8	Cesarean	No
13	Pos	Pos	Pos	Neg	Pos	39/G6P2	1.18	None	None	4, 8	Cesarean	No
14	Pos	Pos	Pos	Pos	Pos	34/G1P0	N/A	9.3	18.4	8, 9	Cesarean	No
15	Pos	Pos	Pos	Pos	Pos	36/G6P5	N/A	None	None	8, 9	Cesarean	No
16	Pos	Pos	Pos	Pos	Pos	39/G1P0	N/A	3.6	19.2	8, 9	Vaginal	No

*Note*: Reference ranges for HB are 15.0–24.0 gm/dL, Bilirubin is 0.3–1.2 mg/dL.

Abbreviations: IAT, indirect antiglobulin test; Pos, positive; Neg, negative; DAT, direct antiglobulin test; Ab, antibody; GA, gestational age in weeks; GP, gravida para; HB, hemoglobin; apgar scores at 1 and 5 min; score of 2 and above (no cyanosis, body and extremities pink); None, no results; N/A, not applicable.

**TABLE 4:** Past medical history and pregnancy complications during gestation of the 16 cases.

Case	Past medical history	Pregnancy complications
1	None	None
2	None	None
3	Chronic Hypertension, Diabetes Mellitus Type 2	Preeclampsia with severe features
4	Systemic Lupus Erythematosus, Antiphospholipid Syndrome	Pregnancy induced hypertension
5	Anemia	None
6	Hepatitis C	None
7	Diabetes Mellitus Type 2	Gestational hypertension
8	None	N/A
9	None	Preeclampsia
10	Anemia	N/A
11	Anemia	None
12	Thrombocytopenia	Preeclampsia with severe features
13	None	None
14	Thrombocytopenia	Gestational Diabetes Mellitus
15	None	Severe preeclampsia
16	Mixed Connective Tissue Disease	None

Abbreviation: N/A, not applicable.

## 5 DISCUSSION

The development of a warm autoantibody during pregnancy is believed to occur very rarely. In our study, we demonstrated the occurrence of warm autoantibody in 16 of a total of 23 510 pregnancies; that is, about 1 in 1500 cases. This contrasts with two other previous studies, Hoppe et al.^7^ where they found warm autoantibodies were significantly more frequent in pregnant women, about 1 in 900 cases when compared to age‐matched non‐pregnant women. Surucu et al.^8^ found warm autoantibodies in 0.08% of pregnant women screened. There is little information about the incidence of warm autoantibody in healthy individuals. Our results are, however, in agreement with these two studies. The prevalence of pregnancy‐induced warm autoantibody was comparable in all three studies (0.1^7^ vs. 0.08^8^ vs. 0.06%). Based on the results of these three studies, pregnancy rarely stimulates the production of warm autoantibodies. Unlike the previous two studies, our study followed the warm autoantibody cases until delivery to assess for HDFN. There was no report of jaundice or HDFN in any of the newborns.

AIHA has rarely been reported in pregnancy. Earlier case reports demonstrated evidence of hemolysis in the fetus during the last week of pregnancy when the mother was diagnosed with AIHA. Baumann and Rubin^9^ reported a case of a patient with a warm AIHA that occurred in the last week of pregnancy. The baby weighed 2,350 grams and had a positive DAT with IgG. Jaundice developed within the first 24 h of life, and bilirubin peaked on the third day of life. Both mother and baby were blood group A RhD positive. The cultures of the newborn's blood and other viral panel markers were negative. The newborn showed a compensated hemolytic process in the first week. Alpha‐methyldopa, which is still used to treat pregnancy‐induced hypertension, is another rare cause of warm autoantibody during pregnancy. For example, a 36‐year‐old mother taking alpha‐methyldopa was diagnosed with AIHA and delivered a healthy male infant at 37 weeks, 6 days gestation. The baby was born with an unremarkable physical examination and no signs of hemolysis throughout the stay in the fetal medicine unit.^10^ Fattizzo and colleagues^6^ report on a retrospective study on the first multicenter international cohort study evaluating AIHA in the setting of pregnancy/puerperium. The inclusion criteria were the diagnosis of AIHA before pregnancy and de novo AIHA, occurring during pregnancy or puerperium. Comparable to our study among 48,615 pregnancies, 20 were diagnosed before pregnancy, 10 had a relapse, and only 13 cases were de novo AIHA (0.03%; 95% CI, 0.005–0.04). One patient with de novo AIHA during pregnancy received transfusion support exclusively during gestation and was treated postpartum. One early miscarriage was reported in a patient with de novo AIHA during pregnancy. Most of the patients they reported on had AIHA before pregnancy. All these patients, including the one de novo case, met the clinical features of AIHA according to the international consensus definition of AIHA.

Although warm autoantibodies in pregnancy appear to be clinically insignificant for both the mother and the baby, unlike those responsible for AIHA, the serological findings are evident in all our 14 cases. According to Ciobanu et al.,^18^ the immunoglobulin rise in the fetal circulation is different between the four types of IgG, with the fastest transfer observed for IgG1. We did not check the IgG isotype; however, IgG3 or IgG1 + IgG3 are most commonly responsible for warm autoantibody.^19^ Das and Mukherjee reported on the influence of immune‐hematological markers on the severity of in‐vivo hemolysis in warm AIHA. IgG3 alone or in combination with IgG1 has increased potential to cause in vivo hemolysis and highly significant (*p* < 0.001) abnormal values of hemolytic laboratory analytes (hemoglobin, bilirubin and LDH). IgG subclasses have a varying affinity for the Fc receptors, with IgG3 having a higher affinity for mononuclear phagocytes.

The detection of either allo‐ or auto‐IgG antibodies in pregnancy initially reflects a risk for both the mother and child. It is important to note that pregnancy‐induced warm autoantibody may remain detectable for a long time, even years after delivery. Two of our cases were still testing positive for the warm autoantibody: Case 11 at 88 weeks and Case 14 at 6 weeks after delivery.

The question to the transfusion medicine physician is the degree of clinical significance of the IgG autoantibodies. The occurrence of these autoantibodies can create a high degree of consternation resulting in recommendations such as repeated testing in the blood bank and hematology laboratories or repeated MCA Doppler studies. Since the conclusion of our study, every pregnant patient with a warm autoantibody without AIHA that we have encountered proceeded to have a healthy pregnancy through delivery. To avoid false alarms and unnecessary investigative procedures, all involved fetal maternal medicine physicians, gynaecologists, transfusion medicine physicians, serologists, and immunohematologists should be informed of the clinically insignificant nature of these autoantibodies. Second, maternal exposure to fetal RBC antigens in the blood during pregnancy may explain how pregnancy may stimulate maternal autoantibody against similar fetal RBC antigens of the mother. Warm autoantibody has been shown to demonstrate specificity for Rh blood group antigens.^12^, ^13^, ^14^ It is interesting in our study to find that every patient with a warm autoantibody was RhD positive while the remaining patients with only alloantibodies were RhD negative. And none of them have clinical features of AIHA.

Measuring the MCA peak systolic velocity by Doppler ultrasonography allows one to estimate fetal haemoglobin in cases with elevated risk of anaemia, with a sensitivity close to 100% and a false positive rate of 12% for severe anaemia. However, severe anaemia is suspected when the MCA systolic peak velocity is ≥1.5MoMs.^18^ None of the four patients with MCA Doppler studies in our study had high systolic peak velocities. Even though there were no clinical features of AIHA in the mothers, MCA Doppler was performed to assess the risk of fetal anaemia in three cases and intrauterine growth restriction in one case. There were no MCA Doppler performed in the remaining 10 pregnancies with warm autoantibody. Given the limitations of MCA Dopplers in predicting fetal anaemia due to potential positives^19^ and reduced accuracy at later gestational ages,^20^ it might be reasonable to incorporate clinical guidance from the Society for Maternal Fetal Medicine. Since current guidelines do not recommend MCA Doppler screening in mothers with only warm autoantibodies,^21^ one must question why these three cases need MCA Doppler studies which can be quite expensive and may be non‐diagnostic in cases of warm autoantibody.

The main limitation of our study was that all the 16 pregnant patients were not evaluated for antibody screen and DAT before pregnancy; even though 10 of them had multiple pregnancies, therefore preexisting serologic abnormalities, if present, could not be entirely excluded. However, all the 16 patients developed the warm autoantibody during pregnancy. They were all de novo positive for warm autoantibody, with no alloantibodies detected.

In conclusion, our study did not show evidence of AIHA or HDFN in any of the mothers. Even though the maternal autoantibody may cross the placenta, the clinical significance of a warm autoantibody in pregnancy remains unknown; our study demonstrates that a warm autoantibody is rare in pregnancy and the autoantibody detected may not be clinically significant. It would appear prudent in cases where the mother has clinical features and severity of warm AIHA during pregnancy to monitor the baby for fetal anaemia.

## AUTHOR CONTRIBUTIONS

**Emmanuel A. Fadeyi:** Conception and design; acquisition, analysis, and interpretation of data; drafting and reviewing the manuscript. Final approval of the version to be published. **Amit Saha:** Analysis and interpretation of data; drafting and reviewing the manuscript; final approval of the version to be published. **Joshua Cox‐Jones:** Conception and design; interpretation of data; drafting and reviewing the manuscript. Final approval of the version to be published. **Daniel Katz:** Conception and design; interpretation of data; drafting and reviewing the manuscript. Final approval of the version to be published. **Bettina Turner:** Acquisition, analysis, and interpretation of data; final approval of the version to be published. **Christina S. Warren:** Conception and design; acquisition, analysis, and interpretation of data; drafting and reviewing the manuscript; final approval of the version to be published. **Gregory J. Pomper:** Conception and design; acquisition, analysis, and interpretation of data; drafting and reviewing the manuscript. Final approval of the version to be published.

## FUNDING INFORMATION

The author(s) received no financial support for the research, authorship, and/or publication of this article.

## CONFLICT OF INTEREST STATEMENT

The authors have no competing interests.

## PATIENT CONSENT STATEMENT

There is a full waiver of consent/assent; therefore, a written informed consent was not required for this research.

## Data Availability

Data can be made available on request.
